# In search of sustainable and inclusive mobility solutions for rural areas

**DOI:** 10.1186/s12544-022-00536-3

**Published:** 2022-04-06

**Authors:** Helen Poltimäe, Merlin Rehema, Janika Raun, Age Poom

**Affiliations:** 1 School of Economics and Business Administration, University of Tartu, Narva mnt 18, 51009 Tartu, Estonia; 2 Stockholm Environment Institute Tallinn Centre, Erika 14, 10416 Tallinn, Estonia; 3 School of Humanities, Tallinn University, Narva mnt 25, 10120 Tallinn, Estonia; 4 Mobility Lab, Department of Geography, University of Tartu, Vanemuise 46, 51003 Tartu, Estonia; 5 Digital Geography Lab, Department of Geosciences and Geography, University of Helsinki, Gustaf Hällströmin katu 2, 00014 Helsinki, Finland; 6 Ruralia Institute, University of Helsinki, Lönnrotinkatu 7, 50100 Mikkeli, Finland

**Keywords:** Rural mobility, Sustainable mobility solutions, Demand-responsive transport, Shared mobility, Public transport, Permanent residents, Temporary residents

## Abstract

**Background:**

Despite emerging research on novel mobility solutions in urban areas, there have been few attempts to explore the relevance and sustainability of these solutions in rural contexts. Furthermore, existing research addressing rural mobility solutions typically focuses on a specific user group, such as local residents, second-home owners, or tourists. In this paper, we study the social inclusivity, economic viability, and environmental impacts of novel mobility solutions in rural contexts based on published scholarly literature. When doing so, we bring both permanent and temporary residents of rural areas under one research framework.

**Methods:**

We used grey literature to identify and categorise novel mobility solutions, which have been applied in European rural areas and are suitable for travelling longer distances. By using six service flexibility variables, we reached four categories of novel mobility solutions: semi-flexible demand-responsive transport, flexible door-to-door demand-responsive transport, car-sharing, and ride-sharing. We analysed the social inclusivity, economic viability, and environmental impacts of those categories based on criteria and evidence identified from scholarly literature by including the perspectives of both permanent and temporary residents of rural areas.

**Results:**

Our findings revealed that while single novel mobility solutions are seldom applicable for all rural travellers, strong spatial and temporal synergies exist when combining different solutions. The need for a connected and flexible set of mobility solutions sensitive to the temporal and spatial patterns of mobility needs is inevitable. Accessible and easily understandable information on routing, booking, and ticketing systems, as well as cooperation, shared values, and trust between various parties, are key success factors for sustainable rural mobility.

**Conclusion:**

Integration of the needs of various user groups is essential when aiming to achieve the provision of environmentally, socially, and economically sustainable mobility solutions in rural areas.

**Supplementary Information:**

The online version contains supplementary material available at 10.1186/s12544-022-00536-3.

## Introduction

Rural areas have traditionally relied on private transport. Long travel distances, low local population density, and the seasonality of temporary residents’ visits to rural areas have created challenges for responding to the travel needs with well-functioning public transport service as a sustainable alternative to private vehicles [1, 2]. However, in the current climate crisis, there is an urgent need for finding and implementing sustainable, i.e., environmentally sound, socially inclusive, and economically viable rural mobility solutions. In Europe, the transport sector accounts for about 25% of total greenhouse gas (GHG) emissions and is the only sector, in which the emissions were increasing until the COVID-19 pandemic [3]. The majority of GHG emissions, but also other external costs of the transport sector, are related to the use of private cars [4].

Transport decisions have been typically made based on traditional economic approaches, including monetary costs and efficiency [5]. However, the primary consideration of economic aspects tends to neglect the social, environmental, and health issues of transport services [6]. Only during recent years, social and environmental considerations have become an important factor in transport-related decision-making, at least in urban areas [7]. Nevertheless, most European countries have not yet developed relevant policies or set clear targets for sustainable rural mobility [8]. Due to the low density of rural areas, the provision of public transport tends to be economically inefficient and enforces the reliance on private cars.

While addressing social inclusiveness in rural transportation, it is important to consider the differences in the needs and possibilities of various user groups, such as the permanent and temporary residents of rural areas. However, there is a lack of research that holistically approaches all rural user groups when assessing mobility solutions for rural areas. In the scholarly literature, the mobility of permanent and temporary residents is discussed in two different strands of literature. One strand focuses on the travel behaviour as well as factors that challenge or support the mobility of permanent residents, such as households with retired people, working-age population, and children (e.g., [9–12]). Another strand of literature focuses on the travel needs and behaviour of domestic and foreign tourists (e.g., [13–16]). Also, second-home owners are traditionally researched under the tourism research paradigm [17]. This separation of scholarly discussion results in a research gap in whether and how rural transport systems can provide mobility solutions that meet the diverse needs of all rural user groups.

Conventional public transport system faces multiple challenges when aiming to respond to the diverse user needs of all traveller groups in rural areas because people have different reasons, abilities, and opportunities to travel, but the system is rather inflexible. The last decade has witnessed an increase in the provision of new, both demand-responsive transport (DRT) and shared mobility solutions in rural areas [8, 18–20], along with similar but more visible counterparts in densely populated urban areas [21]. Although several of the solutions were introduced already in the beginning of the 1970s in North America and UK [22], their implementation, especially in rural areas, has geared up only during the last decade along with the development of information and communication technology [23]. We use the umbrella term “novel mobility solutions” in the remainder of the study to denote the emerging demand-responsive and shared mobility solutions with a common denominator. These novel mobility solutions aim to offer environmentally sound substitutes to GHG-intensive private transportation and improve accessibility to transport and destinations when compared to conventional public transport [18, 23]. Therefore, in this paper, we consider these novel mobility solutions to be environmentally sustainable alternatives to private cars.

Sustainable, connected and flexible set of mobility solutions, which are sensitive to the temporal and spatial patterns of the diverse mobility needs in rural areas, are inevitable. However, much of the scholarly attention paid to novel mobility solutions focuses on urban contexts and the use of smart technologies, such as mobility-as-a-service solutions, smart city applications, or shared mobility models [24–26]. In contrast, mobility solutions relevant for rural contexts, and how they could ensure better, more sustainable, and socially inclusive mobility options for rural travellers, have received only scant attention in the scholarly literature. Furthermore, the economic viability of those solutions to provide continuous and trustworthy mobility services in rural contexts has not received enough systematic attention.

**Fig. 1:**
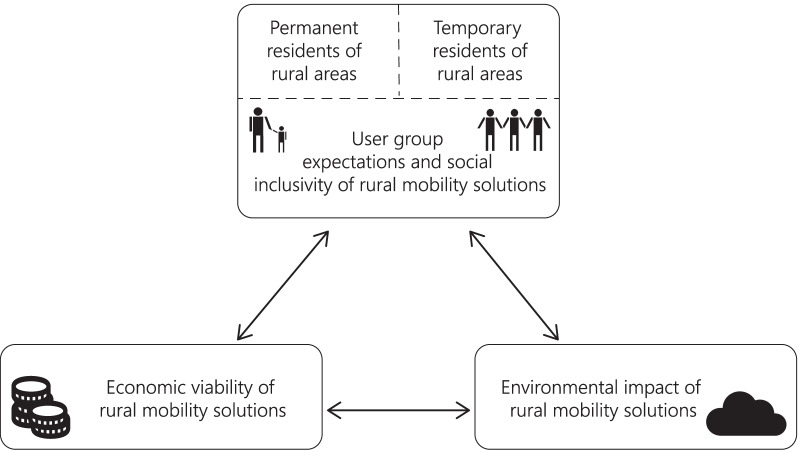
Sustainability issues in the context of rural mobility solutions

In this paper, we study the applicability and sustainability of novel mobility solutions in rural contexts based on scholarly literature. When doing so, we bring both permanent and temporary residents of rural areas under one research framework to identify their expectations to mobility solutions as well as potential synergies and controversies between different user groups. Based on the literature review, we aim to understand the social inclusivity, economic viability, and environmental sustainability of novel mobility solutions in rural contexts (see Fig. 1). According to our knowledge, this has not been done in previous scholarly literature.

## The challenges of sustainable rural mobility

Rural areas suffer from unsustainable mobility solutions for a range of reasons. Rural areas are defined as areas with less than 300 persons per km^2^ [27] and are hence characterised by low population density. Furthermore, due to widespread urbanisation, rural areas often face decreasing and ageing populations [28] alongside the withdrawal of jobs, shops, services, and schools [29, 30]. At the same time, rural areas often function as hinterlands to urban cores to which jobs, education, services, and leisure are concentrated. Low population density and dependence on urban cores result in longer commuting distances in rural areas travelled by fewer people [31], which enforce the reliance on private cars.

Traditionally, private transport has been the dominant mobility solution in areas with low population density [12, 32]. Specifically, older people [33] and households with children [34, 35] have been demonstrated to be reliant on private cars. Similarly, temporary rural residents tend to use private transport to travel to their destination and within the local region while being on holiday [36, 37]. Both convenience and the absence of choice favour the use of private cars, however, the attachment to private transport is also affected by emotional and liberating factors [38] and may have become a local norm [39]. However, private transport as the prevailing mobility solution does not comply with the principles of sustainable mobility due to environmental and equality concerns.

The mobility needs and access requirements among rural population groups vary considerably. Permanent residents travel mainly for work, education, healthcare, maintenance, socialisation, and leisure. While working age adults are considered to be independent travellers, some user groups, such as younger children and older people, may require assistance with travel due to their limited ability and rights to travel independently. The mobility needs of temporary populations include mainly travel to their destination and within the local area for leisure, maintenance, and socialisation purposes. This applies to both second-home owners [2, 37] and tourists (e.g., [40–42]).

Peak visitor numbers among temporary residents occur in summer, on weekends, and on national holidays [43] and have been exemplified also during the Covid-19 pandemic [44]. This may even outweigh the number of permanent residents, especially in popular tourist destinations, scenic regions, and areas with many second-home properties [43, 44]. If the fluctuation in visitor numbers is not accounted for, demand for transport services or infrastructure might be underestimated [17]. However, some transport services designed for local residents may be inaccessible for foreign tourists due to language and information barriers [45, 46]. Thus, the differences in the needs, expectations, and abilities of travellers add further complexity to the provision of inclusive and sustainable rural transport services.

Novel mobility solutions tend to require good access to the internet and skills to use information and communication technology [47]. However, the quality of mobile phone networks and mobile internet varies greatly in rural areas due to low population density, in contrast with urban regions [8, 19, 34]. This hinders the use of digitally assisted transport services. Furthermore, the adoption of those services often requires devices connected to the internet and skills to use the devices and related applications. This may function as a severe barrier to several user groups and hinder the transition towards sustainable mobility solutions [12, 39].

## Methods

**Fig. 2:**
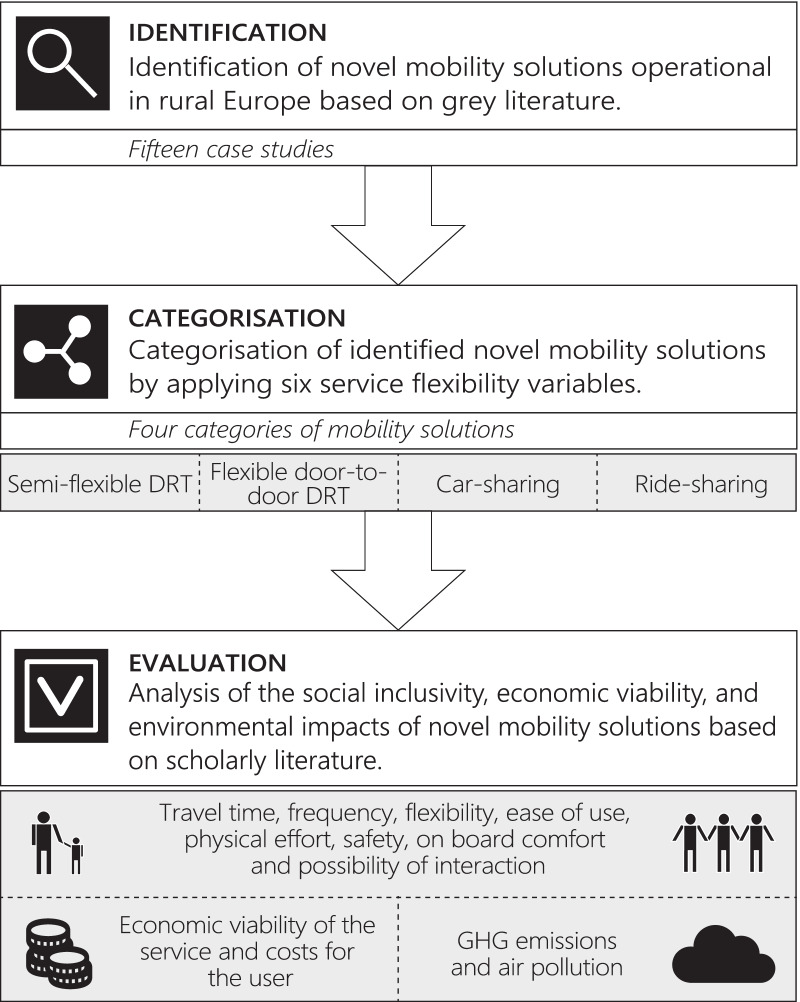
Methodological workflow of the study

We analysed the social inclusivity, economic viability, and environmental impacts of novel mobility solutions in rural contexts based on published scholarly literature. To structure our literature review and subsequent analysis, we first used grey literature to identify and categorise novel mobility solutions, which have been already applied in European rural areas and are suitable for travelling longer distances. This approach left out solutions that are better adaptable in urban regions, such as bike-sharing or e-hailing. We categorised identified mobility solutions based on six service flexibility variables, which are described in Sect. 3.2. This resulted in four categories of novel mobility solutions: semi-flexible demand-responsive transport, flexible door-to-door demand-responsive transport, car-sharing, and ride-sharing. In the next step, we used these mobility categories as analytic units to evaluate their sustainability based on published research evidence. Specifically, we searched for scholarly literature that addressed the social, economic, and environmental aspects of mobility solutions in rural contexts. This enabled the evaluation of the applicability and sustainability of each category from the perspective of both permanent and temporary residents. The methodological workflow of this study is provided in Fig. 2 and elaborated further in the below sections.

### Identifying novel mobility solutions for rural areas

New adaptive mobility solutions, which are emerging in both rural and urban contexts, have been often defined as (a) ‘demand-responsive transport’ (DRT) or ‘flexible transport’ [48] and (b) ‘shared mobility solutions’ or ‘shared transport’ [8, 19]. The terminology is ambiguous and not fully developed, resulting in a diverse use of terms in the scholarly literature and everyday use. DRT denotes a service that lies between fixed regular public transport and personalised taxi services [49], depends largely on public financing, and may offer flexibility in terms of route choice, trip scheduling, on-demand stops, etc. [11]. According to Wang et al. [50], public transport can be considered DRT if it is available to the general public, it is provided by low-capacity road vehicles (small buses, vans, or taxis), the route and/or timetable can be altered, and the fare is charged per passenger. Shared mobility, on the other hand, is part of the concept of the sharing economy and related business models are typically developed through private initiatives. It can denote bike-sharing, car-sharing, car-pooling, or ride-sharing [19, 24] and the fees are generally charged per vehicle. While DRT is considered to be the key solution to the contemporary challenges of rural mobility, shared mobility is seen to complement conventional public transport [8].

Although rural mobility is increasingly on the international research agenda, there is a lack of scholarly literature providing evidence of the performance and operational phase of novel rural mobility solutions. However, several EU projects focusing on rural mobility have been launched over the past ten years, such as MAMBA,^1^ MARA,^2^ G-PaTRA,^3^ Peripheral Access,^4^ SMARTA,^5^ and RESPONSE.^6^ As the outcomes of these projects have rarely been discussed in academic journals, we relied on ‘grey literature’ when identifying the mobility solutions operational in rural areas. Specifically, we used the reports from the EU-funded projects RESPONSE [51] and SMARTA [52], which provided a systematic overview of existing sustainable rural mobility solutions. In addition, we gathered information from the websites of relevant transport operators involved in these projects. In total, we identified fifteen case studies representing novel mobility solutions operational in European rural areas. The cases are presented in the Additional file 1 and were used for the categorisation of different types of novel mobility solutions in rural areas.

### Categorisation of novel mobility solutions

We categorised identified mobility solutions to reach analytical categories for the following sustainability evaluation based on scholarly literature. For the categorisation, we applied six flexibility variables, which are considered most relevant both in the studied project reports [51, 52] and in the previous scholarly literature [11, 19, 20, 53, 54]. Namely, we analysed the services based on (1) route, (2) stop, and (3) scheduling variables describing the network typology and flexibility between fixed, semi-flexible, and flexible door-to-door services. Also, we included service aspects, such as (4) booking requirements, (5) sharing the vehicle with other riders, and (6) the type of vehicle into the categorisation (see Table 1 and the Additional file 1).

**Table 1:** Categorisation of novel rural mobility solutions based on service flexibility variables

	Route	Stops	Scheduling	Booking	Ride-sharing	Vehicle type	Example cases from RESPONSE and SMARTA projects
Conventional public transport	Fixed	Fixed, can be skipped	Fixed	Not required	Shared	Bus, minibus	
Designated tourist buses	Fixed	Fixed	Fixed	Required	Shared	Bus	
Semi-flexible DRT	Fixed, semi-flexible*	Semi-flexible**	Fixed, flexible***	Required	Shared	Bus, minibus, car	Flextrafik (Denmark) TFI Local Link (Ireland) Elba island MaaS (Italy) Kylakyyti (Finnland) Tornio (Finland) Anropsstyrd trafik (Sweden) Bus Alpin (Switzerland) Connect2Wiltshire (UK)
Flexible door-to-door DRT	Flexible	Flexible	Flexible	Required	Shared, private	Minibus, car	GO-MOBIL (Austria) Flextrafik (Denmark) TFI Local Link (Ireland) Elba island MaaS (Italy) Bummelbus (Luxemburg) Bravoflex (Netherlands) HentMegSauda (Norway) Connect2Wiltshire (UK)
Car-sharing	Flexible	Semi-flexible****	Flexible	Required	Private	Car	Talbont Energy (Wales)
Ride-sharing	Semi-flexible	Semi-flexible****	Semi-flexible	Required	Shared	Car	REZOPOUCE (France)

*A route with pre-defined stops that may be skipped and served in a flexible order depending on current demand

**Stops may be predefined along a route or in an area and may be skipped and served in a flexible order depending on current demand

***Services that operate both fixed and demand-based schedules depending on the time of day or week

****Depending on pick-up and drop-off locations

Most of the identified mobility solutions provided an alternative or complementary transport service to existing public transport. The services functioned as the ‘last leg’ of a trip, often also referred to as ‘last-mile services’, in areas with low population density or infrequent scheduled services or in areas, which are located far from existing service networks. Most studied transport services used a mixed service model, which did not follow discretely any of the defined variable options. This is because the operators modify the flexibility of routing and scheduling, and the vehicle type depending on local needs and business opportunities within the service area. Some mobility solutions provided transport services similar to regular public transport with fixed-route, stops, and timetables, also for seasonal demand. Others provided fully flexible door-to-door services up to providing a digital platform for hitchhiking. We identified no clear association between service flexibility and targeted user group; all options from fixed-route fixed-stops scheduled services to flexible transport were used for the broad range of transport users, from daily commuters to tourists and temporary residents.

Almost all studied services required booking of the service beforehand, with a temporal range from at least 30 min to the previous day, either online or by telephone. Several services also required user registration and identification before booking or using the service. The prevailing vehicle type used in the studied cases was a minibus. However, some operators used cars for routes or time slots with low user rates. This means that although the services studied aimed to provide a shared transport service, at times some of them functioned similarly to a private taxi service. On the other hand, the operators also used regular buses in case of higher demand.

Based on the six service variables, we divided the identified DRT and shared mobility services into four simplified categories to assess their social inclusivity from the perspective of different user groups as well as their economic viability and environmental impacts. Table 1 presents both the service variables and the resulting categories: semi-flexible DRT, flexible door-to-door DRT, car-sharing, and ride-sharing, with examples from the analysed case studies. In addition, we used conventional public transport and designated tourist buses as reference values in the analysis.

### The sustainability evaluation of novel mobility solutions

We used scholarly literature to identify significant factors affecting social inclusiveness and user group expectations, economic viability, and environmental impacts of rural mobility solutions and analysed the sustainability of four categories of novel rural mobility solutions based on those factors. We searched for relevant literature in the scholarly databases Scopus, Web of Science, and Google Scholar. We used a combined literature search strategy including both keyword search and snowball method of cited research, by applying a broad set of keywords. The literature analysis resulted in the following set of sustainability-related factors of rural mobility solutions. Under social inclusiveness and user group expectations, the prevailing keywords were travel time, frequency, flexibility, ease of use, physical effort, safety, onboard comfort, and the possibility of interaction. Social inclusiveness was evaluated from the perspectives of both permanent and temporary user groups. Under economic aspects, we focused on the economic viability of the service and the costs for the user. Under environmental issues, the main related keywords in the scholarly literature considering rural contexts were GHG emissions and air pollution. The production, use, and disposal of vehicles cause also other impacts [55, 56]. However, the lifecycle perspective falls out of the scope of this paper, because it is mainly related to the vehicle, not the specific mobility solution. We analysed the economic and environmental issues in comparison to the use of private cars and did not differentiate user group needs here.

## Analysis

### Social inclusivity and user group expectations of novel mobility solutions

#### Travel time and frequency

Optimal time use has been considered one of the main reasons for preferring cars to other modes of transport. Private cars are thought to provide higher levels of independence, freedom, and control over time, therefore being even more attractive when time appears to be short [38, 57]. The longer the travel time in comparison to private cars, the less attractive public transport becomes [11, 39], even with lower prices [58]. For permanent residents, it is important how public transport schedules are aligned with specific commitments (e.g., to facilitate commuting) and how various modes of transport interconnect [34].

The frequency of public transport and waiting time are important considerations for permanent residents [11, 34], but also for second-home owners, who have raised this as one of the main reasons to prefer private over public transport [2]. The car-reliance of second-home owners is also related to the need to carry items, such as food, laundry, or waste [2].

Time-related factors, such as journey length, schedules, or waiting and booking times, have also been found to be important to tourists in rural destinations [15, 59, 60]. In an urban context, it has been shown that flexibility, comfort, and speed of mobility contribute to the competitiveness of the destination among tourists: the better the public transport, the more attractive the destination is to tourists [14]. This might be applicable also in rural contexts. At the same time, tourists using coach services are unlikely to spend much time within an area [42].

#### Flexibility

It has been found that time flexibility provided by DRT attracts more frequently these rural inhabitants who travel for work [50]. But it is also denoted as a general tendency that local residents prefer more flexible mobility solutions [11].

As for tourists, they often prefer private cars because these offer freedom and flexibility, and tourism itself is an escape from usual time-bound regimes [57]. Easy access to tourist destinations and the possibility and freedom to plan one’s journey independently are important factors for tourists [61].

#### Ease of use

Easily understandable information on scheduling and routing available for people with different digital competency levels is one of the main prerequisites for using public transport, DRT, and shared mobility options [11, 15, 39, 62]. Easy access to information seems to be especially important for tourists, who have no prior knowledge of local transport opportunities [61]. The need to understand the details of the local network, various ticketing options, or the locations of stops may increase the perceived risk factor of DRT [40]. If tourists have some previous experience with the destination [14] or if they have experienced problems in finding parking space [63], they might be more prone to use alternative modes of transport to private vehicles.

Many DRT and ride-sharing options are accessible only via specific platforms for registered users [64, 65], which may function as a barrier for tourists. Similarly, pre-registration requirements of DRT may be another entry barrier for both tourists and infrequent local users [19]. In the case of ride-sharing, the information of available mobility options is often shared via a local community or specific user group [64]. This indicates that these services are most probably not designed for wider use and are hardly accessible for (foreign) tourists.

Integrated, multimodal, and multi-operator ticketing system offers convenience and flexibility for all user groups [66]. Some European cities have introduced so-called ‘destination guest cards’ that often come free with booked accommodation and entitle users to free public transport usage among other benefits [13, 67]. By reducing the budget requirements for the entire time spent in the destination, such offers not only shape transport choices but can also represent a unique selling proposition that influences destination choice [13, 40].

#### Physical effort and safety

Due to dispersed settlement, total travel distances and the distance to the closest public transport stop are generally longer in rural than in urban areas. The degree of physical effort and agility required to undertake a journey are important factors shaping the travel choice. Distance to the nearest stop and the need to change vehicles have the greatest effect on perceived accessibility: the longer the walking distance to the stop and the more changes are needed, the less public transport is preferred, especially among older people [1, 39, 68]. At the same time, an adequate transport system could encourage walking and contribute to the better physical health of older people [33]. As demonstrated by Hansen et al. [69], rural residents have a higher probability of being overweight and obese due to the lack of possible active transportation, compared to the residents of urban areas. Accessibility is also related to safety: if the trip to a bus stop is perceived to be dangerous, e.g., in terms of traffic intensity on the way to a public transit stop or the need to cross a busy road, rural residents may prefer to use cars [34].

The propensity to use shared mobility solutions is affected by the distance to shared vehicles and the uncertainty regarding the location at which the vehicles can be collected and returned [25]. Regarding ride-sharing, it has been challenging to attract sufficient vehicles to the service regardless of demand [64]. Furthermore, reluctance to trust new transport services and adapt travel behaviour may hinder the use of ride-sharing solutions, especially among older people [70]. Some studies have highlighted that ride-sharing is perceived as dangerous if the driver is not familiar with the user [34].

#### On-board comfort and possibility of interaction

Aspects of on-board comfort are related to cleanliness, safety, space, and onboard amenities, such as Wi-Fi, screens, food, and drinks. These affect travel experience, especially on medium- and long-haul trips [71, 72]. On longer trips to destination, tourists and second-home owners have been reported to prefer public transport over private cars to spend time on more pleasurable activities than driving [2, 40].

DRT and shared mobility solutions have been shown to increase social inclusion in rural areas by providing more equal access to public transport and destinations, especially for people with no access to cars [23, 65]. In addition, they increase social contacts and interaction between local residents and other travellers, provide opportunities to enjoy the scenery during travel, avoid the stress of driving in unfamiliar locations, and take part in local social activities [60, 62, 66, 72, 73]. In the case of tourists, their interests and willingness to spend time in local surroundings and money on local services differ greatly and are at least to some degree related to the travel mode [41, 42].

Table 2 outlines the social considerations of conventional public transport, designated tourist buses, and the four categories of novel mobility solutions comparatively from the perspectives of permanent and temporary residents. In short, the quality of conventional public transport is perceived to be poor because of limited or no flexibility in routing, stops, or scheduling, low frequency, and long travel time. Different DRT solutions offer flexibility, but there might be a trade-off between flexibility and the size of vehicles. Flexible door-to-door DRT, car-sharing, and ride-sharing provide the greatest flexibility, but their limiting factor is the possibility to match demand and supply for various user groups at preferred times. Ride-sharing, meanwhile, may be perceived as dangerous, due to unfamiliar drivers. For tourists, barriers to shared mobility solutions include access to comprehensible information and restrictions associated with certain payment schemes.

**Table 2:** The advantages and disadvantages of different mobility solutions for permanent and temporary residents in rural areas

	Permanent residents		Temporary residents	
	Advantages	Disadvantages	Advantages	Disadvantages
Conventional public transport	+ fixed stops and routes are easy to understand	− no flexibility in routing, stops, or scheduling − typically, low frequency − typically, long travel time − typically, stops are not close to all users − not convenient for people with poor health (physical effort required) − scheduling cannot consider all user groups and purposes (commuting, healthcare, groceries, etc.)	+ fixed stops and routes are easy to understand + good opportunities to communicate with locals	− no flexibility in routing, stops, or scheduling − routes and stops not designed according to tourist needs − typically, long travel time − ticket information might not be well available for tourists
Designated tourist buses		− cannot be used by local residents	+ on−demand service + optimal travel and short waiting time + price might remain unnoticed in a full cost model	− not adaptive to individual interests − fixed times, limited frequency − fixed routes, no flexibility − no opportunities to communicate with locals − limited opportunities to spend time and money on local services
Semi-flexible DRT	+ shorter travel time + additional connections to stops or areas assigned according to local residents needs + flexibility depends on specific DRT solution, can be provided on different levels + cost not very high	− availability depends on predefined service area − smaller vehicles may not cover the demand	+ higher frequency than traditional public transport + shorter travel time + flexibility depends on specific DRT solution, can be provided on different levels + good opportunities to communicate with locals + cost not very high + can be organised to be in line with second-home owners’ destinations	− availability depends on predefined service area − only semi-flexible travel planning possibilities − smaller vehicles may not cover the demand − ticket information might not be well available for tourists
Flexible door-to-door DRT	+ as frequent as necessary + short travel time + possibility to wait at the origin point + very flexible + preferred by groups who prefer one-seat trips	− depends on the availability of vehicles − may become more expensive	+ as frequent as necessary + short travel time + possibility to wait at the origin point + very flexible + can be organised to be in line with second-home owners’ destinations	− depends on the availability of vehicles − may become more expensive − ticket information might not be well available for tourists
Car-sharing	+ as frequent as necessary + possibility to arrange own trip + short travel time + privacy + very flexible + typically, not very high costs	− depends on the availability of cars (which usually is a challenge) − in destination, might be issues with parking	+ as frequent as necessary + possibility to arrange own trip + short travel time + privacy + very flexible + typically, not very high cost	− depends on the availability of cars − in destination, might be issues with parking − information about the cost and availability of cars is not easy to find for tourists (usually, in the local language)
Ride-sharing	+ short travel time + flexible, if supply and demand are balanced (which usually is a challenge) + typically, not very high cost	− availability depends on similar travel routes − safety issues with an unknown driver	+ short travel time + flexible, if supply and demand are balanced (which usually is a challenge) + possibility to get to know local people + typically, not very high cost	− availability depends on similar travel routes − difficult to match the needs of different user groups − information about the cost and availability of cars is not easy to find for tourists (usually, in the local language) − fee system might be set up in a way not suitable for tourists (platform fee)

Compiled from information provided in the following studies: Cass et al. [91], Guiver et al. [62], Le-Klähn and Hall [15], Graham et al. [33], Berg and Ihlström [34], Martín Martín et al. [61], Morsche et al. [11], Næss et al. [2], Scuttari et al. [60], Cottrill et al. [1], Juschten and Hössinger [40], Orsi et al. [59] and Lygnerud and Nilsson [64]

### Economic viability of novel mobility solutions

#### Economic viability

The economic viability of public transport related business models is far more complex in rural than in urban contexts [12]. Larger-scale businesses are more robust because these engender economies of scale and related competitive advantages, although smaller local companies are often more community-minded and well-perceived among residents [74]. For any public transport solution, the availability of financial resources from various stakeholders is a crucial success factor [10]. Rural transport including tourism-related mobility solutions requires financial and policy support from local and national governments [1, 40]. Approaching the total mobility need and the range of mobility solutions provided in a given region as a whole and thus eliminating fragmentation between transport agencies, service providers, and within ticketing and route planning services for users is an important element of success for sustainable rural mobility [53].

Greater flexibility often means higher operational costs. For example, in the case of a taxi-based scheme Regiotaxi, one passenger-kilometre costs for the government seven times more than one passenger-kilometre on scheduled public transport [52]. At the same time, larger scales of implementation of flexible mobility services reduce the costs and subsidy requirement per passenger [20, 68]. By using a simulation-based analysis, Kim [75] showed that the fares of a door-to-door DRT solution should not exceed 50% of a taxi service to be attractive and socio-economically feasible for users. The lower the population density, the higher the need for passenger subsidy [20].

Revenue streams for the online platforms of shared mobility solutions are fragile: the perception of the applications as free or very low-cost decreases the willingness to pay for the service [76]. As a result, people are willing to pay less than the costs of providing the service are [74]. Another important success factor is the ‘matchmaking’ quality as it affects the number of community members registered [77]. Also, the lack of control over vehicles and the insufficient supply of vehicles at certain times have been highlighted as factors affecting the use of shared mobility solutions [64].

#### Costs for the users

The fares of public transport are defined on a personal basis, while the cost per passenger decreases when sharing a car [40]. The cost-efficiency of car use is an important aspect among second-home users, who typically take their whole family on a trip [2]. Several researchers have argued that cost is a significant barrier to public transport use [1, 34, 78], and higher prices reduce the attractiveness of this alternative to private cars [11]. It has also been demonstrated that the willingness of tourists to replace a private car with public transport is affected by cost [59, 60].

The willingness to pay for shared mobility solutions is very low due to the perception of a free service. In addition, tariff schemes for car-sharing services are considered to be very rigid, i.e., these are not flexible and tend not to have user-specific features [25].

Table 3 outlines the main advantages and disadvantages of different mobility solutions from the economic viability viewpoint. As demonstrated by various studies, greater flexibility comes with increasing costs spent on the provision of the service. Traditionally, local or national authorities have been major contributors to public transport or flexible DRT solutions, but the need to contribute from the user end is increasing. For shared mobility models, financial success is difficult to ensure because users’ willingness to pay for such services is low.

**Table 3:** The economic viability of different mobility solutions when compared to private car or tourist rental car

	Advantages	Disadvantages
Conventional public transport	+ low user costs compared to private car when travelling alone	− no cost reduction per person when travelling with several people (scale effect from car use) − if cost is perceived as high, barrier to use − major funding needed from local/national authorities
Designated tourist buses	+ minor funding needed from local/national authorities	− typically, needs a higher contribution from the user − coach travellers don’t spend much time and money on local products and services
Semi-flexible DRT	+ the larger the implementation scale, the lower the cost	− if user costs are perceived high, a car is preferred − higher cost of the system compared to traditional public transport due to flexibility − major funding needed from local/national authorities − costs should be divided between different stakeholders
Flexible door-to-door DRT	+ the larger the implementation scale, the lower the cost	− typically, needs a higher contribution from the user − higher costs of the system compared to traditional public transport due to flexibility − major funding needed from local/national authorities
Car-sharing and ride-sharing	+ minor funding needed from local/national authorities	− high expectations for the service, but low willingness-to-pay − difficult to ensure a sustainable business model − very dependent on matchmaking quality and ensuring a sufficient number of cars

Composed based on Baker [68], Mullay and Nelson [78], de Jong et al. [10], Pronello and Camusso [76], Guyader and Piscicelli [77], Cottrill et al. [1], Panzer-Krause [42], Porru et al. [12], SMARTA [52] and Lygnerud and Nilsson [64]

### Environmental impact of novel mobility solutions

#### GHG emissions and air pollution

In travel mode comparison, the highest GHG emissions are related to private car use, followed by public transport, while walking and conventional cycling are not related to GHG emissions [79–82]. The actual impact is influenced by service frequency, total mileage covered, occupancy rate, vehicle type, and fuels used [65, 83]. Replacing regular, fixed public transport with DRT options reduces GHG emissions due to decreased mileage and hardly any ‘empty running’ of buses,the total effect may increase when alternative fuels are used [65, 83]. Replacing private cars with DRT of a higher occupancy rate per vehicle decreases GHG emissions [23]. The DRT cases considered in this paper have shown a consistent increase in user numbers over time, which has been related to a positive environmental effect. For example, the assumed reduction in car use between 2005 to 2011 in the case of the Alpine Bus service was shown to result in a total net saving of 100 tons of CO_2_ [52].

The use of shared mobility models has shown to decrease GHG emissions compared to private car [84], however, so far, the effects have only been evaluated in urban, not rural, contexts. The effect size depends largely on occupancy rate, although in the longer term positive environmental effects might decrease due to eventual rebound effects, such as people travelling more [79, 85]. Furthermore, if a private car is substituted for a taxi-like service, where a single passenger is picked up, or a minibus is used for single passenger transport, the effect on emissions might become adverse [20, 78]. The environmental advantages and disadvantages of different mobility solutions in rural contexts are outlined in Table 4.

**Table 4:** Environmental impacts of different mobility solutions compared to private car or tourist rental car

	Advantages	Disadvantages
Conventional public transport and designated tourist buses	+ decrease in GHG emissions compared to private transport, assuming sufficient occupancy rate	− the potential positive effect might not be realised, if low occupancy
Semi-flexible and flexible door-to-door DRT	+ decrease in emissions dependent on occupancy rate and vehicle/fuel used + lower vehicle ownership rate	− the more flexibility, the smaller effect on emissions
Car-sharing and ride-sharing	+ small decrease in emissions (dependent on occupancy rate and vehicle/fuel used) + lower vehicle ownership rate	− due to rebound effect, the potential might not be realised

Composed based on Mullay and Nelson [78], Firnkorn and Müller [85], Ryley et al. [65], Reichert et al. [81], Ferrero et al. [25], Amatuni et al. [79], Coutinho et al. [83], Jochem et al. [26] and TNMT [82]

## Discussion and conclusions

*Previous studies of rural mobility have focused on distinct user groups instead of a holistic approach to all users of rural transport.* Furthermore, there is only scant research evidence of the seasonal travel demand of various types of tourists, their expectations and perceptions of rural mobility services, and the suitability of local public transport solutions for them. Also, real-life cases identified from grey literature tend to be designed primarily for permanent residents. A holistic approach to developing mobility services that serve all user groups could bring economic synergies because of the temporal differences in the peak travel demand and wiser use of the vehicle fleet (see [13]). For example, while the peak demand among permanent residents falls on school and work days, temporary rural residents require transport services more during weekends and holidays. A more widespread provision of DRT solutions could meet the needs of both permanent and temporary residents. For example, fixed or semi-flexible DRT solutions could serve both user groups on routes with overlapping interests. Public transport services with partially fixed routes can serve specific tourist destinations with steady travel demand, such as ski resorts and national parks. Door-to-door services provide almost the same level of flexibility as taxis, with significant potential for users in areas with less regular travel patterns. The best solution depends on the local context, the type and seasonality of tourism, the location of the main tourist attractions, and the interests and background of tourists. For example, DRT might not be the best option for large and time-limited peaks in demand, for which designated buses might serve tourists better.

*A connected and flexible set of mobility solutions sensitive to the temporal and spatial patterns of mobility needs are inevitable.* So far, the diverse needs of user groups have triggered the provision of a range of parallel mobility solutions in rural areas, as a single mobility solution cannot fit the needs of all users [68, 74]. Furthermore, rural areas rely heavily on private transport. However, the ways how to integrate different mobility solutions in rural contexts to achieve synergies both from the perspective of service providers and users have not deserved sufficient research attention. The solutions could involve conventional public transport integrated with door-to-door or small-scale mobility solutions [10, 19, 68] or with other types of transport services, such as school, healthcare, or shopping transport [34]. A thorough spatiotemporal analysis of the needs and interests of various user groups and the (misalignments of) current transport service provision when developing mobility services in rural areas has the potential to reduce the reliance on private cars and related environmental load, achieve greater economic efficiency, and improve access to transport services.

*Serving the mobility needs of tourists with local public transport would create several benefits for rural areas.* Integrated solutions would yield in more funds and higher occupancy rates for public transport, better opportunities for interaction between locals and tourists, and a greater likelihood that tourists spend money on local goods and services outside touristic ‘hot spots’. A common client pool with improved access for tourists also improves accessibility for locals, such as people working in tourist locations, implying a reduction in transport poverty and increased social inclusion [1, 39]. Furthermore, integrated solutions may be designed to address specific needs of both user groups, such as the need to transport large items. For example, tourists may need space for luggage, sports equipment, or bikes [47], while locals may need access and space for wheelchairs or pushchairs as well as for bikes. Lack of these options causes a barrier for using the service and a missed opportunity to reach sustainable mobility solutions for rural areas.

*A broader perspective linking the social, economic, and environmental issues of rural mobility solutions can address internal controversies.* While highly flexible mobility solutions both in terms of timing and stops/routes are preferable from the perspectives of both permanent and temporary residents, these solutions imply low occupancy rates. Hence, highly flexible solutions are likely to be less viable economically and bring less benefits for the environment, therefore a combination and integration of different mobility approaches is needed. Other controversies include aspects related to the economic interests of different service providers, safety issues of the service, or required levels of digital skills to use novel mobility solutions (e.g., [70]).

*The potential of car-sharing and ride-sharing is yet to be realised in rural areas.* This is evident from the lack of scholarly literature about successful implementation of car-sharing or ride-sharing solutions in rural areas. Also, only two out of fifteen real-life cases identified from grey literature used shared mobility solutions. Shared mobility solutions could decrease car dependency among rural households [19, 64]. As substitutes to private cars, the use of shared solutions also signals that people increasingly understand and follow the car-as-a-service model [25, 26]. Ride-sharing services provide more opportunities to arrange daily mobility for residential user groups, and these could also be suitable for seasonal residents and tourists who stay longer in the region. Specifically, ride-sharing services could replace typical rental services and provide a modern way of hitch-hiking with additional long-distance connection opportunities. A new strand of scholarly literature discusses the opportunities that autonomous vehicles may provide for a range of user groups [23, 86]. However, their applicability in rural contexts is rarely elaborated and involves a risk of exclusion due to a large proportion of the senior population.

*Cooperation, shared values, and trust between various parties are key success factors.* for sustainable rural mobility both in case of shared mobility solutions [74] and public transport services [10, 87]. Cooperation to serve tourist travel needs with public transport may result in optimised resource use and higher occupancy rates but requires willingness and dedication from all involved parties. For example, the lack of interest among tourism companies hindered the implementation of a free public transport system for tourists in German holiday destinations, despite strong support from public transport authorities and transport companies [13]. Thus, in tourist regions, collaboration between tourism operators, transport operators, public administration as well as tourists, local commuters, and other residents is needed [76].

*Data generated from the use of mobility solutions should be available for research and decision-making while safeguarding individual privacy.* These large data sets reflect mobility needs and behaviour of current user groups, offer new opportunities for service development, and enable a better understanding of the social, economic, and environmental implications of transport solutions [88]. However, due to data privacy and ethical concerns [89], accessing, processing, and disseminating the data need clear set of rules and transparent methodologies.

*Accessible and easily understandable information on routing, booking, and ticketing systems is universally important for all user groups* (see [15, 19]). Saying this, preferences for information channels and formats may vary among users. For example, tourists require information at the time of booking their trip: information of travel options to the area as well as within the area should be part of the overall marketing of the area, preferably in a range of languages and in both digital and printed formats. Specific concerns may arise regarding the digital competencies of users and the quality of digital infrastructure in rural areas. Decent spatial coverage and a stable provision of the internet are critical success factors for the uptake of novel mobility solutions in rural areas [8].

*The transition to novel mobility solutions that serve both permanent and temporary residents requires well-planned policies*, which are lacking for rural areas [8]. These should be increasingly targeted at enhancing access to public transport, improving the energy efficiency of mobility solutions, and managing the fleet of privately-owned vehicles [90]. The need to restrict private car use also includes tourist travel in scenic areas [59, 60]. To achieve these transport policy aims without strong adverse effects on the attractiveness of the region, alternative mobility solutions and policies supporting their uptake must be put in place.

*Future research on rural mobility should address local contexts, user needs and travel behaviour, and the provision of transport services holistically.* Understanding the accelerators and barriers of the uptake of novel mobility solutions in rural contexts is critical to enhancing the sustainability of rural mobility solutions. Furthermore, mobility solutions should be carefully inspected in terms of their social, economic, and environmental implications, including fair access to transport and destinations as well as data privacy issues. This study has demonstrated that DRT and shared mobility solutions are promising from both environmental and social perspectives and could meet the needs of various user groups if designed properly. However, the willingness of rural user groups to adapt to novel mobility offers requires more scholarly attention as the current research has remained limited and fragmented in terms of spatial and sociodemographic coverage. Qualitative data and mixed methods approach would be needed to better inform us about the social inclusiveness of novel mobility solutions in rural regions. Another limiting factor for the wider adoption of novel solutions is the common struggle about economic efficiency while ensuring a price that meets users’ expectations. Nevertheless, several operational transport services have shown that careful planning can result in the successful application of novel mobility solutions in rural areas (see, e.g., [51]).

## Supplementary Information

**Additional file 1.** Examples of rural mobility solutions implemented in European rural areas.

## Data Availability

Not applicable.

## Footnotes

1 https://www.mambaproject.eu/.

2 https://www.mara-mobility.eu/.

3 https://northsearegion.eu/g-patra#.

4 https://www.interreg-central.eu/Content.Node/Peripheral-Access.html.

5 https://ruralsharedmobility.eu/.

6 https://response-project.eu/.
