# Supplementary material for: In search of sustainable and inclusive mobility solutions for rural areas
Source: Eur Transp Res Rev. 2022 Apr 6;14(1):13. doi: 10.1186/s12544-022-00536-3 (PMC8983330; doi:10.1186/s12544-022-00536-3)
Supplement: Supplementary file 1 — Additional file 1. Examples of rural mobility solutions implemented in European rural areas. [file 12544_2022_536_MOESM1_ESM.docx]

**Additional file 1.**

Examples of rural mobility solutions implemented in European rural areas and their attributes based on the analysis of DRT cases active in 2020 in the RESPONSE [51] and SMARTA [52] projects.

| DRT case/  Country | Route flexibility | | | Origin-Destination service | | | | Scheduling | | | Booking | | Shared/private ride | | Vehicle type | | |
| --- | --- | --- | --- | --- | --- | --- | --- | --- | --- | --- | --- | --- | --- | --- | --- | --- | --- |
|  | Fixed | Semi-flexible | Flexible | Fixed stops | Stops flexible along route | Stops flexible in an area | Door-to-door | Fixed | Semi-flexible | Unscheduled | Required | Not required | Shared | Private | Bus | Minibus | Car |
| GO-MOBIL  Austria |  |  | x |  |  | x | x |  |  | x | x |  | x | x |  | x | x |
| Flextrafik  Denmark |  | x | x |  |  | x | x |  | x |  | x |  | x |  |  | x | x |
| Kylakyyti  Finland |  | x |  |  | x | x |  |  | x |  | x |  | x |  |  | x |  |
| Tornio  Finland | x |  |  |  | x |  |  | x |  |  | x | x | x |  | x |  |  |
| REZOPOUCE  France |  |  | x |  |  | x | x |  |  | x | x |  | x |  |  |  | x |
| TFI Local Link  Ireland |  | x | x |  |  | x | x | x |  |  | x |  | x |  | x | x |  |
| Elba island MaaS platform, Italy | x | x | x | x | x | x | x | x | x | x | x | x | x | x | x | x | x |
| Bummelbus  Luxemburg |  |  | x |  |  | x |  |  |  | x | x |  | x |  |  | x |  |
| Bravoflex  Netherlands |  |  | x |  |  |  | x |  |  | x | x |  | x | x |  | x | x |
| HentMegSauda  Norway |  |  | x |  |  |  | x |  | x |  | x |  | x | x |  | x |  |
| Anropsstyrd trafik  Sweden | x |  |  |  | x |  |  | x |  |  | x |  | x |  |  |  |  |
| Bus Alpin  Switzerland | x | x |  | x | x |  |  | x |  |  | x | x | x |  | x | x |  |
| Connect2Wiltshire  UK | x | x | x |  | x | x | x | x | x | x | x |  | x |  |  | x | x |
| DaRT  UK |  |  | x |  |  | x | x |  | x |  | x |  | x |  |  | x |  |
| Talybont Energy  Wales |  |  | x |  |  | x |  |  |  | x | x |  |  | x |  |  | x |
